# Mechanism of Water-Enhanced Volatile Aldehyde Release in Oil Fumes from Thermal Oxidation of Oleic Acid: Insights from Synchrotron Radiation Photoionization and Gas Chromatography–Mass Spectrometry

**DOI:** 10.3390/molecules31040594

**Published:** 2026-02-09

**Authors:** Bing Qian, Xuan Zhu, Chulian Su, Hongxing Li, Qiong Wu, Chengyuan Liu, Yang Pan, Bingjun Han

**Affiliations:** 1 Analysis and Test Center, Chinese Academy of Tropical Agricultural Sciences; Key Laboratory of Quality and Safety Control for Subtropical Fruit and Vegetable, Ministry of Agriculture and Rural Affairs; Key Laboratory of Tropical Fruits and Vegetables Quality and Safety, State Administration for Market Regulation; Hainan Provincial Key Laboratory of Quality and Safety for Tropical Fruits and Vegetables; Key Laboratory of Nutritional Quality and Health Benefits of Tropical Agricultural Products of Haikou City, Haikou 571101, China; qianbinglss@163.com (B.Q.); zhuxuan0223@163.com (X.Z.); suchulian1015@163.com (C.S.); lihongxing_2018@163.com (H.L.); joan627@126.com (Q.W.); 2 National Synchrotron Radiation Laboratory, University of Science and Technology of China, Hefei 230029, China; 3 College of Food Science and Engineering, Hainan Tropical Ocean University, Sanya 572022, China

**Keywords:** cooking oil fumes, water, oleic acid, acetaldehyde, acrolein

## Abstract

Thermal oxidation of edible oils during high-temperature cooking produces complex fumes containing harmful volatile compounds. However, the role of water, a common co-reactant in practical cooking, remains insufficiently understood. In this study, oleic acid was used as a model compound to investigate thermal oxidation. Online monitoring using synchrotron radiation photoionization mass spectrometry (SR-PIMS) revealed that water significantly increased the emission of volatile acetaldehyde and acrolein, with maximum increases of 164% and 123% at 10% water addition. Complementary offline GC-MS analysis showed enhanced formation of (*E*)-2-decenal, (*E*,*E*)-2,4-decadienal, and (*E*)-2-undecenal, suggesting these unsaturated aldehydes may be key intermediates. Mechanistically, oleic acid underwent free radical-mediated peroxidation to form (*E*)-2-decenal, (*E*)-2-undecenal, and (*E*,*E*)-2,4-decadienal. These intermediates subsequently decomposed into acetaldehyde and acrolein via hydration, retro-aldol condensation, and hydroperoxide scission, with water accelerating both processes. Overall, these findings highlight water’s critical role in promoting the generation of harmful volatile aldehydes in oil fumes.

## 1. Introduction

High-temperature cooking induces thermal oxidation of edible oils, generating complex oil fumes comprising volatile aldehydes, ketones, hydrocarbons, furans, and others. These compounds affect food flavor and may pose health risks [1]. In China, oil fumes are a major source of household indoor air pollution [2], contributing to lung cancer in non-smoking women [3,4] and increasing the risk of respiratory infections and tuberculosis among professional chefs [5]. Among the various aldehyde pollutants in oil fumes, acrolein and acetaldehyde have attracted considerable attention due to their high volatility, significant toxicity (including the ability to cross-link with proteins, disrupt biological functions, and damage DNA) [6], and widespread presence in emissions. Acrolein is listed by the Agency for Toxic Substances and Disease Registry and the U.S. Environmental Protection Agency as a high-priority toxic chemical and air pollutant [7,8]. Acrolein and acetaldehyde are classified by the International Agency for Research on Cancer as Group 2A and 2B carcinogens, respectively [9].

Edible oils primarily consist of triglycerides (>95%), formed by glycerol esterified with three fatty acids. During heating, triglycerides hydrolyze, releasing free fatty acids that undergo further oxidation to generate oil fumes [10]. Due to the complexity of real edible oils, model fatty acids are often used for mechanistic studies. Oleic acid, a dominant unsaturated fatty acid in vegetable oils, is widely adopted as such a model [11,12,13]. In practical cooking, water is inevitably introduced through food or cooking processes. Previous studies indicate that water accelerates triglyceride hydrolysis and increases oil fume emissions [14,15]. However, its specific influence on aldehyde release during oleic acid oxidation and the underlying mechanism remain insufficiently understood.

GC-MS is commonly used for oil fume analysis but has limitations: sampling containers (e.g., SUMMA canisters [16], Tedlar bags [17]) may deactivate reactive species during storage, and sorbent-based [18] or SPME [19] sampling requires time-consuming pretreatment. GC-FID/MS also struggles with C1~C3 aldehyde detection, while 2,4-DNPH derivatization (for GC-MS [20], LC [21] and LC-MS [6]) is hazardous, complex, and prone to analyte loss. Critically, these offline methods cannot capture dynamic chemical evolution. Proton transfer reaction (PTR)-MS [22,23] and secondary electrospray ionization (SESI)-MS [24] enable direct real-time detection but rely on exact mass identification and cannot differentiate isomers with distinct toxicological profiles (e.g., acetone [16] and propanal [17] in real oil fumes).

Photoionization mass spectrometry (PIMS) offers soft ionization near analyte ionization thresholds, yielding clear molecular ions with minimal fragmentation. Compared to VUV discharge lamps and lasers, synchrotron radiation (SR) light provides high photon flux, stability, and tunable energy, which enables precise differentiation of isomers based on their ionization energies and makes it ideal for PIMS. Our prior work [25] demonstrated that SR-PIMS can comprehensively characterize complex oil fumes from oleic acid thermal oxidation and continuously monitor their evolution for 55 h, underscoring its potential for such studies.

This study employed oleic acid as a model compound for vegetable oils and combined online SR-PIMS with offline GC-MS to systematically investigate the promoting effect of water on the formation of harmful aldehydes, particularly acetaldehyde and acrolein, during oleic acid thermal oxidation. Compared with conventional offline sampling methods, SR-PIMS enables the first in situ, real-time tracking of the dynamic evolution of these aldehydes under the condition of adding water midway. By correlating key precursor species, potential reaction pathways involving water were inferred. These findings clarify the critical role of water in oil fume formation, providing direct experimental evidence for understanding the origin of fume toxicity in real cooking scenarios and offering insights for strategies to reduce harmful emissions during daily cooking.

## 2. Instruments and Methods

### 2.1. Reagents

Oleic acid (analytical reagent grade, AR) was purchased from Aladdin Biochemical Technology Co., Ltd. (Shanghai, China). Ultrapure water (18.2 MΩ·cm) was obtained from a Merck Millipore Synergy UV system (Merck KGaA, Darmstadt, Germany). High-purity synthetic air (80% N_2_ and 20% O_2_) was supplied by Nanjing Special Gas Co., Ltd. (Nanjing, China).

### 2.2. Synchrotron Radiation Beamline Parameters

The SR-PIMS experiments were conducted at the BLU3U beamline of the National Synchrotron Radiation Laboratory (NSRL) in Hefei, China. This beamline is equipped with three TOF-MS endstations arranged coaxially and separated by vacuum valves. All three endstations utilize synchrotron radiation for photoionization and are dedicated respectively to combustion studies [26], catalysis research [27], and molecular characterization [28]. In this study, the characterization-focused TOF-MS endstation was employed. The beamline parameters are briefly summarized as follows [26]: Gratings with line densities of 200 and 400 lines/mm provided tunable photon energies of 5–14 eV and 10–21 eV, respectively. The photon flux reached 10^13^ photons/s at a beam current of 400 mA, and the energy resolution was 4000. An argon-filled gas filter suppressed higher-order harmonic radiation.

### 2.3. Instruments for SR-PIMS

**Figure 1:**
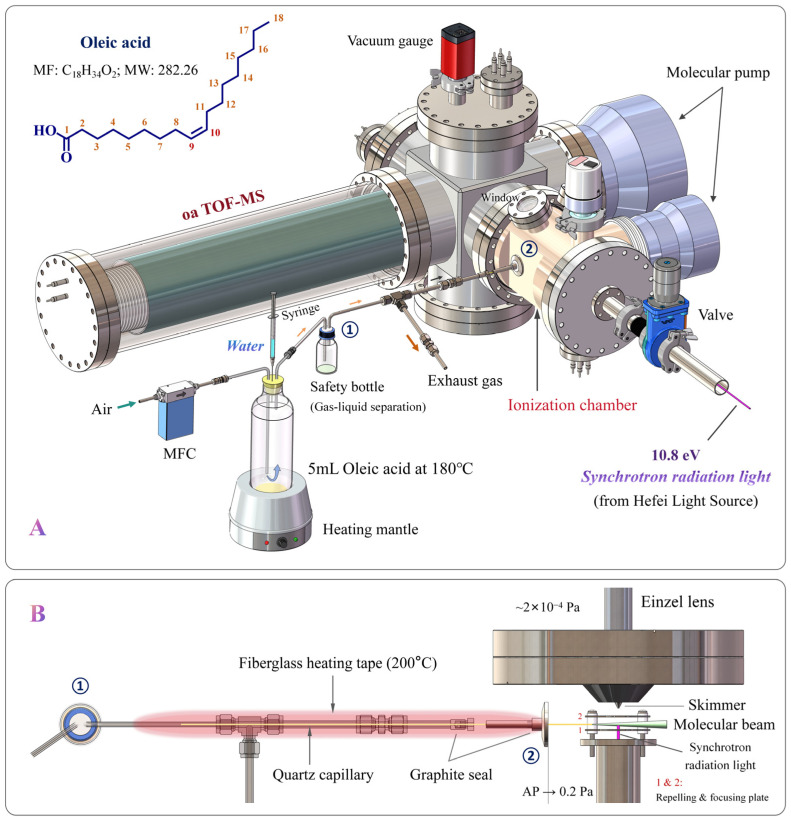
(**A**) Schematic of the SR-PIMS setup for online monitoring of volatile fumes from thermal oxidation of oleic acid in the presence of water. (**B**) Illustration of the sampling quartz capillary and the ionization chamber. Note: ① and ② in panels (**A**) and (**B**) are for rapid positioning.

The schematic of the on-line SR-PIMS setup is shown in Figure 1A. The pressure of the homemade TOF-MS ionization chamber was ~0.2 Pa, and that of the mass analyzer chamber was ~2 × 10^−4^ Pa. A fused quartz sampling capillary (OD 360 μm, ID 250 μm, length 45 cm; Lianyungang Aokai Quartz Co., Ltd., Lianyungang, China) connected the ionization chamber to the atmosphere (Figure 1B). Driven by the pressure difference, gaseous analytes were continuously drawn in to form a neutral molecular beam at the capillary outlet, which intersected with the VUV SR light. When the photon energy slightly exceeded the analyte’s ionization energy, the molecular ions ([M]^+^) were generated via soft ionization.

Ions were transferred from the ionization chamber to the mass analyzer through an ion guide system comprising coaxial repelling and focusing electrodes, a nickel skimmer (1 mm orifice), and an einzel lens. The capillary outlet was positioned near the center of the electrodes. The skimmer enabled differential pumping and improved ion transmission, while the einzel lens focused the ions through a slit into the orthogonal acceleration (oa) region. The ions were separated by their *m*/*z* values in the flight tube and detected by a microchannel plate (MCP) detector. Data acquisition and processing were performed using a custom LabVIEW 2018 program.

Additional devices included a mass flow controller (MFC; MKS Instruments, Inc., Andover, MA, USA), an electric heating mantle (Yancheng Loikaw Technology Co., Ltd., Yancheng, China), and a fiberglass heating tape (Taizhou Junqian Electric Heating Equipment Co., Ltd., Taizhou, China) with a temperature controller (Yuyao Yitai Instrument Factory, Yuyao, China).

### 2.4. Instruments for GC-MS

Offline GC-MS analysis was performed using an Agilent 8890 gas chromatograph (Agilent Technologies, Inc., Santa Clara, CA, USA) coupled with a 5977B mass spectrometric detector. An HP-5 capillary column (30 m × 250 μm × 0.25 μm) was used with helium as the carrier gas at 1.0 mL/min. The injector temperature was 250 °C, and samples were introduced in split mode (split ratio 10:1, split flow 10 mL/min). The oven temperature was held at 36 °C for 15 min, then ramped at 10 °C/min to 250 °C and held for 20 min. The temperatures of the electron ionization (EI) ion source and the quadrupole were 230 °C and 150 °C, respectively. Mass spectra were recorded over an *m*/*z* range of 35–400 (threshold 150). Data were acquired and processed using Agilent MassHunter 10.0, and compounds were identified by matching spectra with the NIST MS Search 2.3 library.

### 2.5. Experimental Procedure for SR-PIMS

As shown in Figure 1A, 5 mL of oleic acid was placed in a ~500 mL glass bottle and thermally oxidized at 180 °C (optimal frying temperature [17]). An air flow (2000 sccm) was continuously supplied to maintain an oxidative atmosphere, refresh and dilute volatile compounds at the oil–air interface, and transfer oil fumes to the quartz capillary inlet. The capillary, enclosed in a stainless-steel tube wrapped with heating tape and maintained at 200 °C, prevented condensation and clogging. A fraction of the fumes entered the mass spectrometer, while the remainder was exhausted. SR-PIMS continuously monitored volatile species, with each mass spectrum accumulated for 60 s at 10.8 eV photon energy.

During thermal oxidation, various volumes (0.1, 0.25, 0.5, and 1 mL) of ultrapure water were injected using a 1 mL medical syringe. At 180 °C, oleic acid and its semi-volatile oxidation products partially evaporated and were carried by airflow. To prevent contamination and signal attenuation of the mass spectrometer, a 100 mL glass bottle was in the transfer line as a trap for these compounds. SR-PIMS continuously monitored volatile species, with each mass spectrum accumulated for 60 s at 10.8 eV photon energy.

### 2.6. Experimental Procedure for GC-MS

Oleic acid (2 mL) was placed in an open glass vial (30 mL) exposed to ambient air, to which 0 μL (control), 40 μL, or 100 μL of purified water was added and thoroughly mixed. The mixtures were then heated in an oil bath at 140 °C for 1 h, then diluted 100-fold with methanol and analyzed by GC-MS.

## 3. Results and Discussion

### 3.1. Identification of Acetaldehyde and Acrolein

**Figure 2:**
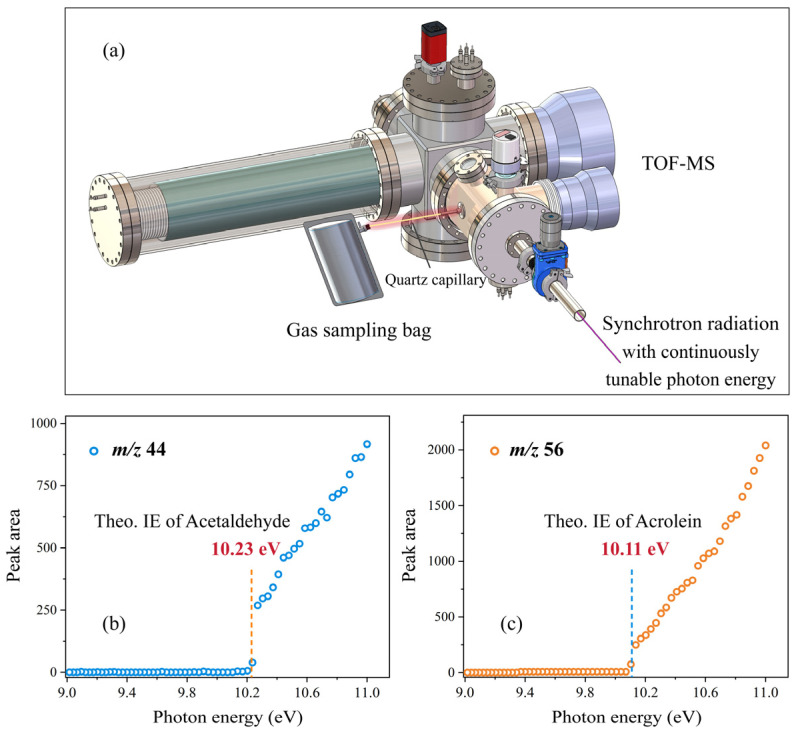
(**a**) Schematic of SR-PIMS analysis of species in the gas sampling bag. Peak area vs. photon energy variation curves for *m*/*z* 44 (**b**) and *m*/*z* 56 (**c**).

Leveraging the tunable photon energy of SR-PIMS, both precise *m*/*z* value matching and energy-dependent analysis were performed for ions at *m*/*z* 44 and 56. Oil fume components were continuously introduced from a gas sampling bag via a quartz capillary (Figure 2a), with photon energy varied programmatically and spectra accumulated for 30 s per energy point. Peak area–photon energy curves were constructed (Figure 2b,c). The signal onsets matched the reference ionization energies of acetaldehyde (10.23 eV) and acrolein (10.11 eV) from the NIST database [29], confirming *m*/*z* 44 and 56 as acetaldehyde and acrolein, respectively.

### 3.2. Water Enhanced Acetaldehyde and Acrolein Release

**Figure 3:**
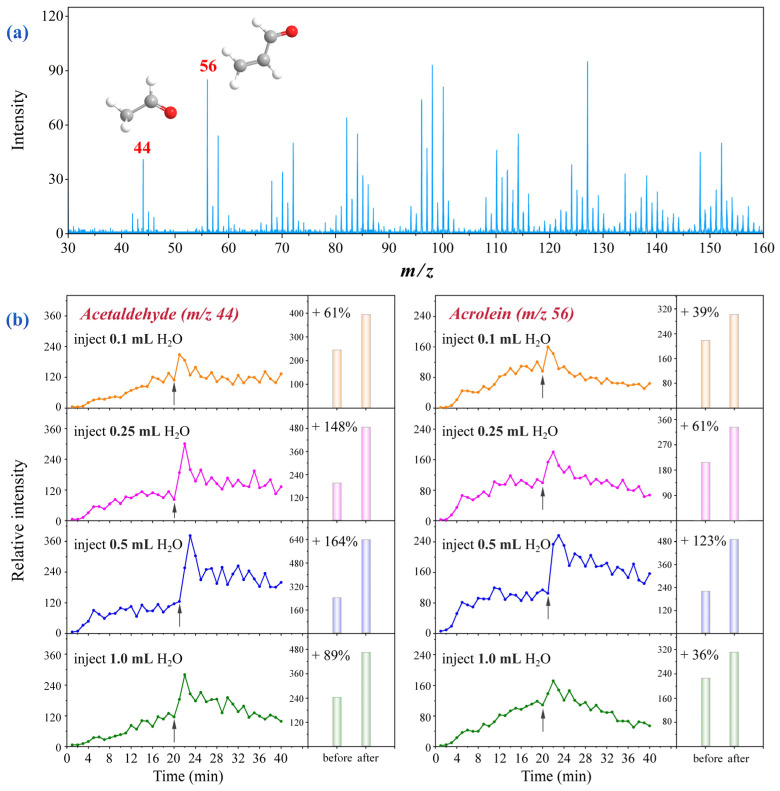
(**a**) Representative SR-PIMS spectrum of volatile products from thermal oxidation of oleic acid (photon energy: 10.8 eV). (**b**) Time-resolved curves and bar charts of acetaldehyde (**left**) and acrolein (**right**). Initial temperature: 50 °C; heating 5 mL oleic acid to 180 °C took ~10 min; held at 180 °C for ~10 min before water injection (arrow). Bar charts: cumulative intensity 2 min pre/post water addition.

The SR-PIMS spectrum (Figure 3a) indicated that the fumes generated from thermal oxidation of oleic acid are complex. This study focused on the effects of water on acetaldehyde and acrolein due to their volatility, carcinogenicity, and high inhalation risk during cooking. Additionally, in the SR-PIMS setup, high-molecular-weight, low-volatility compounds were intentionally minimized to prevent them from entering the mass spectrometer to delay contamination of the ion transfer system.

Time-resolved profiles of acetaldehyde (*m*/*z* 44) and acrolein (*m*/*z* 56) over 40 min are shown in Figure 3b. The temperature increased from 50 °C to 180 °C within ~10 min, and water was injected at the 20-min mark. Both compounds increased steadily during heating, indicating that prolonged oil heating elevates exposure risk. Adding water during the thermal oxidation of oleic acid increased the formation of acetaldehyde and acrolein, and the magnitude of this enhancement first increased and then decreased with rising water content. The strongest enhancement occurred with 10% water (0.5 mL), which increased acetaldehyde and acrolein emissions by 164% and 123%, respectively. Excessive water addition weakened this effect due to heat absorption during water evaporation, thereby lowering the oil temperature and slowing the oxidation reactions.

### 3.3. Water Promoted (E)-2-decenal and (E,E)-2,4-decadienal Formation

The online SR-PIMS experiments were conducted at 180 °C for two reasons. First, 180 °C is widely recognized as the optimal frying temperature [17], making the results more relevant to real cooking conditions and human health. Second, adding water at this temperature induces vigorous reactions, allowing clearer observation of transient changes in acetaldehyde and acrolein. However, preliminary GC-MS tests showed that water presence at 180 °C caused violent reactions and sample splashing, compromising accuracy. Therefore, subsequent GC-MS experiments were performed at 140 °C with less water to ensure stable conditions and minimize sample loss. Although the GC-MS conditions were milder, both methods involved thermal oxidation of oleic acid–water mixtures, and thus may have shared similar reaction characteristics. The GC-MS data remain valuable for elucidating the mechanism of water-promoted acetaldehyde and acrolein formation.

**Figure 4:**
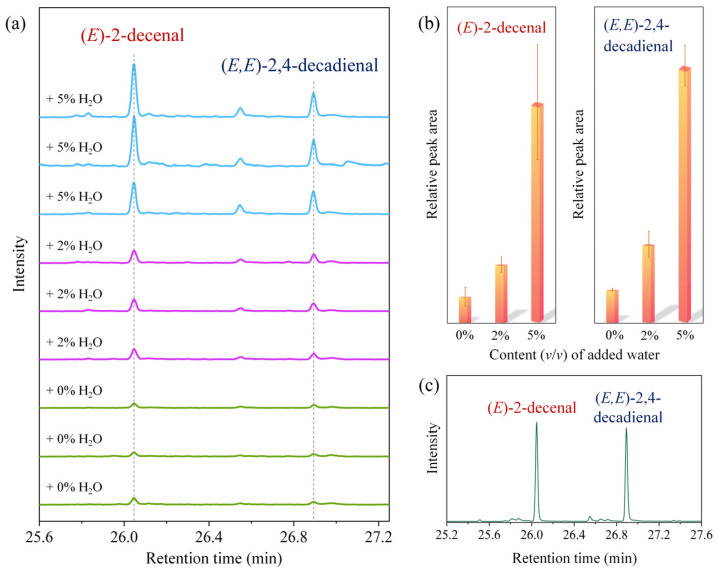
GC-MS chromatograms (**a**) and corresponding peak area bar charts (**b**) of (*E*)-2-decenal and (*E*,*E*)-2,4-decadienal from the thermal oxidation of oleic acid. (**c**) Chromatogram of 5 ppm standard solution.

Our previous characterization of the fumes from the thermal oxidation of oleic acid confirmed the presence of (*E*)-2-decenal and (*E*,*E*)-2,4-decadienal [25]. The current GC-MS results indicated that water promoted their formation, as shown in Figure 4a,b. Upon addition of 2% water, the signal responses of (*E*)-2-decenal and (*E*,*E*)-2,4-decadienal increased by 122% and 135%, respectively; with 5% water, the increases reached 738% and 670%. In addition to NIST database identification, 5 ppm standard solutions of (*E*)-2-decenal and (*E*,*E*)-2,4-decadienal were used to confirm the retention times of the chromatographic peaks (Figure 4c).

### 3.4. (E)-2-decenal as a Key Intermediate

During the thermal oxidation of oleic acid, the presence of water promoted the release of acetaldehyde and acrolein, likely by facilitating the formation of intermediate species such as (*E*)-2-decenal and (*E*,*E*)-2,4-decadienal. (*E*)-2-decenal originates from the primary oxidation product 9-hydroperoxide (9-ROOH) and acts as a potential precursor of (*E*,*E*)-2,4-decadienal [30,31].

**Figure 5:**
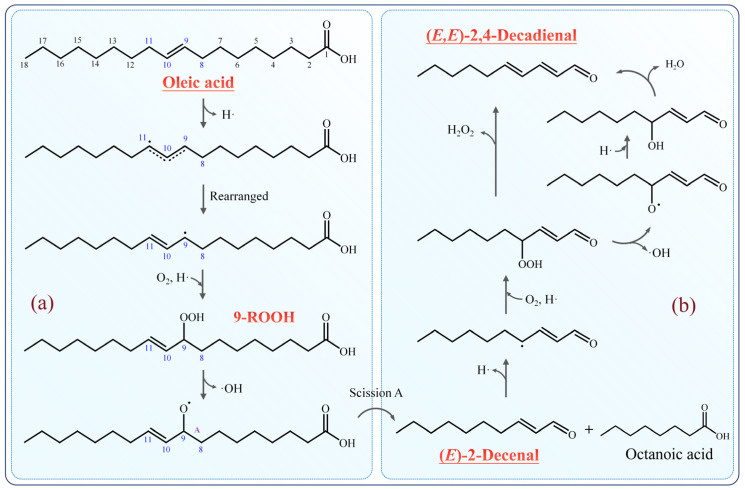
Proposed mechanisms for the formation of (**a**) (*E*)-2-decenal from oleic acid and (**b**) (*E*,*E*)-2,4-decadienal from (*E*)-2-decenal. Note: Oleic acid’s actual configuration is the Z isomer, as shown in Figure 1A.

Oleic acid, a monounsaturated fatty acid, contains a double bond at the C9 position (relative to the carboxyl group), as shown in Figure 5a. The hydrogen atoms adjacent to the double bond (at C8 and C11) are more reactive and readily abstracted compared to those attached to the double-bonded carbons (C9 and C10). Consequently, oleic acid initially loses a hydrogen atom at C11, followed by electron rearrangement, forming a new double bond between C10 and C11 to yield a 9-lipid alkyl radical. This radical reacts with O_2_, abstracting a hydrogen atom to form the primary oxidation product 9-hydroperoxide (9-ROOH). Hydroperoxides are unstable and readily decomposes at room temperature, with their degradation accelerating at elevated temperatures. Specifically, the peroxy bond (i.e., the O-O bond in R-O-OH) of 9-ROOH undergoes cleavage, releasing a hydroxyl radical and forming a 9-alkoxy radical (9-RO·). The 9-RO· intermediate then undergoes homolytic A-scission at the C8-C9 bond, producing (*E*)-2-decenal and octanoic acid.

As shown in Figure 5b, (*E*)-2-decenal loses a hydrogen atom at the allylic methylene carbon via a classical free radical mechanism, undergoing hydroperoxidation to form a hydroperoxide group at this site. Further loss of hydrogen peroxide may generate a new double bond between C4 and C5, potentially forming (*E*,*E*)-2,4-decadienal, with H_2_O_2_ possibly released as a byproduct. Alternatively, the hydroperoxide group may decompose to a hydroxyl-containing compound, which subsequently dehydrates to yield (*E*,*E*)-2,4-decadienal. Increased water content elevates H_2_O_2_ levels, and as a strong oxidant, H_2_O_2_ enhances lipid radical formation during the chain initiation stage, thereby accelerating the thermal oxidation of oleic acid.

### 3.5. (E,E)-2,4-decadienal as a Key Intermediate

**Figure 6:**
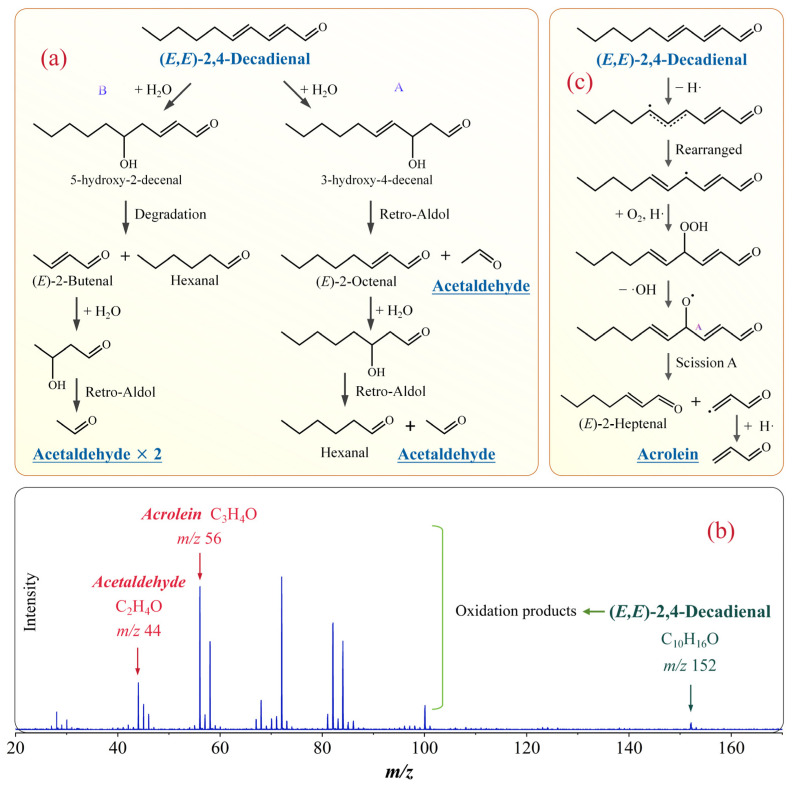
Proposed mechanisms for the formation of (**a**) acetaldehyde and (**c**) acrolein from (*E*,*E*)-2,4-decadienal. (**b**) SR-PIMS spectrum of volatile products from the thermal oxidation of (*E*,*E*)-2,4-decadienal at 180 °C (photon energy: 11 eV).

(*E*,*E*)-2,4-decadienal possesses conjugated double bonds and an aldehyde group, rendering it highly reactive and prone to further reactions. As shown in Figure 6a, under aqueous conditions, (*E*,*E*)-2,4-decadienal undergoes decomposition via double-bond hydration and retro-aldol condensation reactions. In pathway A, (*E*,*E*)-2,4-decadienal first undergoes hydration at the C2=C3 double bond to form 3-hydroxy-4-decenal, which subsequently undergoes retro-aldol degradation to yield (*E*)-2-octenal and acetaldehyde. Similarly, (*E*)-2-octenal further degrades to produce hexanal and another acetaldehyde molecule. In pathway B, (*E*,*E*)-2,4-decadienal undergoes hydration at the C4=C5 double bond to form (*E*)-5-hydroxy-2-decenal, followed by cleavage of the C4-C5 bond, generating hexanal and (*E*)-2-octenal. Subsequently, (*E*)-2-octenal undergoes retro-aldol condensation to produce two acetaldehyde molecules.

In addition, the SR-PIMS spectrum of volatile products from the thermal oxidation of (*E*,*E*)-2,4-decadienal was obtained (Figure 6b). A 5 μL standard sample was heated at 180 °C for 10 min in a ~500 mL glass bottle, and the headspace volatiles were collected with a 20 mL syringe. The quartz capillary of the mass spectrometer was then inserted into the syringe for sampling at a photon energy of 11.0 eV. Based on the accurate *m*/*z* values, acetaldehyde (*m*/*z* 44) and acrolein (*m*/*z* 56) were identified as oxidation products of (E,E)-2,4-decadienal.

The possible mechanism for the formation of acrolein from (*E*,*E*)-2,4-decadienal is illustrated in Figure 6c. Initially, (*E*,*E*)-2,4-decadienal undergoes hydrogen abstraction at the C6 position, followed by electron rearrangement that forms a new double bond between C5 and C6, generating an alkyl radical. This radical subsequently reacts with O_2_, forming a peroxyl radical that abstracts a hydrogen atom and produces a hydroperoxide (ROOH). The hydroperoxide is unstable, and cleavage of the peroxy bond releases a hydroxyl radical, forming an alkoxy radical (RO·). The RO· species then undergoes homolytic A-scission at the C3-C4 bond, yielding (*E*)-2-heptenal and acrolein.

### 3.6. (E)-2-Undecenal as a Key Intermediate

**Figure 7:**
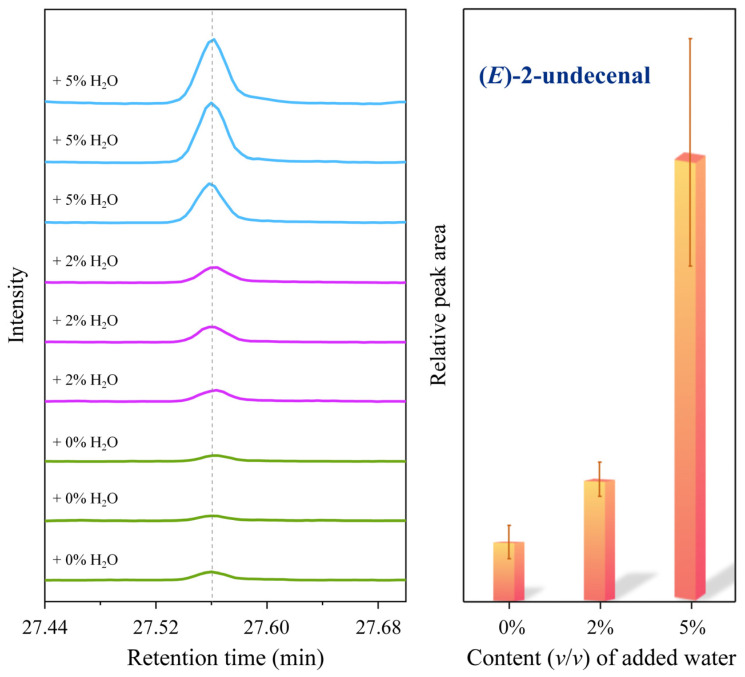
GC-MS chromatograms and corresponding peak area bar charts of (*E*)-2-decenal from the thermal oxidation of oleic acid.

In addition to the aforementioned (*E*)-2-decenal and (*E*,*E*)-2,4-decadienal, GC-MS analysis (Figure 7) revealed that water significantly promoted the formation of (*E*)-2-decenal. With the addition of 2% and 5% water, the yield of 2-undecenal increased by 105% and 653%, respectively. (*E*)-2-decenal was identified by NIST mass spectral matching.

**Figure 8:**
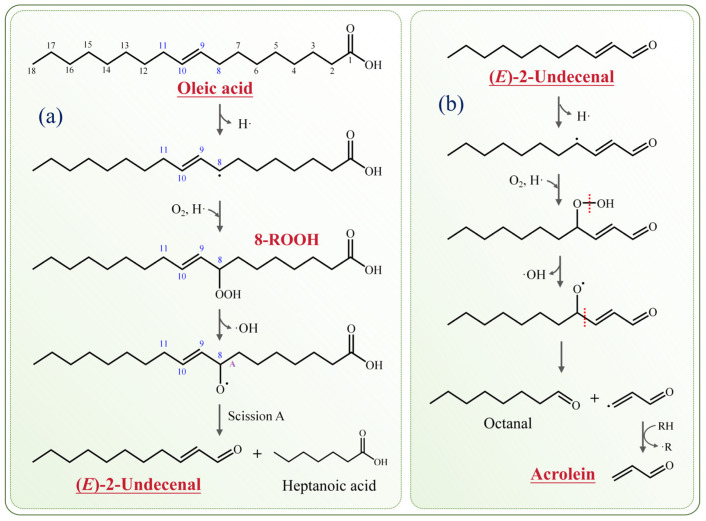
Proposed mechanisms for the formation of (**a**) (*E*)-2-undecenal from oleic acid and (**b**) acrolein from (*E*)-2-undecenal. Note: Oleic acid’s actual configuration is the Z isomer, as shown in Figure 1A.

Based on the classical free radical chain reaction mechanism, (*E*)-2-decenal is speculated to originate from the oleic acid 8-hydroperoxide (Figure 8a). Oleic acid possesses a double bond at the C9 position, rendering the hydrogen atoms on the allylic methylene groups (i.e., C8 and C11) highly reactive and prone to abstraction. Hydrogen abstraction at C8 generates an 8-alkyl radical (8-R·), which subsequently reacts with O_2_ to produce an 8-peroxyl radical (8-ROO·). The 8-ROO· abstracts a hydrogen atom from an adjacent oleic acid molecule, yielding 8-hydroperoxide (8-ROOH). The unstable 8-ROOH undergoes homolytic cleavage of the peroxide bond, releasing a hydroxyl radical and forming an 8-alkoxy radical (8-RO·). Subsequently, the 8-RO· intermediate undergoes homolytic A-scission at the C7-C8 bond, producing (*E*)-2-decenal and heptanoic acid.

Unsaturated aldehydes contain C=C double bonds, making them chemically reactive and prone to further oxidative degradation. Figure 8b illustrates a possible mechanism for the formation of acrolein from (*E*)-2-decenal. The hydrogen atoms adjacent to the double bond are more reactive; thus, the hydrogen at the C4 position of (*E*)-2-decenal is more susceptible to abstraction, forming an alkyl radical. This radical then reacts with O_2_ and abstracts a hydrogen atom to generate a corresponding ROOH. ROOH is unstable and undergoes homolytic cleavage of the peroxy bond to yield an alkoxy radical (RO·). RO· undergoes homolytic β-scission at the C3-C4 bond to produce octanal and acrolein. Based on the same mechanism, the oxidative degradation of (*E*)-2-decenal yields heptanal and acrolein.

This study observed that the contents of the oleic acid thermal oxidation products (*E*)-2-decenal, (*E*,*E*)-2,4-decadienal, and (*E*)-2-undecenal increased significantly after the addition of water, suggesting that these species may serve as key reaction intermediates. On this basis, a potential reaction pathway for their further conversion into acetaldehyde and acrolein was proposed. It should be noted that, first, (*E*)-2-octenal, (*E*)-2-butenal, (*E*)-2-heptenal, hexanal, octanal, and heptanal involved in the proposed pathway have all been identified as thermal oxidation products of oleic acid in our previous study [25]. Second, owing to the complexity of the reaction mechanism, many secondary intermediate species have not yet been subjected to detailed experimental investigation. Therefore, the reaction pathway proposed in this study represents a reasonable inference based on the available experimental results and literature reports and involves a certain degree of uncertainty; further comprehensive experiments are required to refine the mechanism.

## 4. Conclusions

During the actual cooking process, water is inevitably present and may substantially influence the formation of harmful components in cooking oil fumes. This study utilized oleic acid as a model compound to investigate the effect of water on its thermal oxidation products and the underlying mechanisms. Online monitoring via SR-PIMS revealed that moderate water addition markedly enhanced the emission of acetaldehyde and acrolein. Complementary GC-MS analysis further demonstrated that water significantly promoted the formation of (*E*)-2-decenal, (*E*,*E*)-2,4-decadienal, and (*E*)-2-undecenal. These unsaturated aldehydes are proposed to possibly act as key intermediates in the water-induced release of acetaldehyde and acrolein. Mechanistic analysis suggests that water accelerates the lipid free radical chain oxidation process, thereby facilitating the formation of these unsaturated aldehydes, which subsequently decompose into acetaldehyde and acrolein through hydroperoxide formation and cleavage, hydration, and retro-aldol condensation pathways. These findings elucidated the mechanistic relationship between water and the generation of harmful aldehydes in cooking oil fumes. Given that oleic acid represents a major fatty acid in most vegetable oils, the results provide valuable insights into the influence of water on oil oxidation during practical cooking and offer a theoretical reference for mitigating health risks associated with cooking fume exposure.

## Data Availability

The original contributions presented in this study are included in the article. Further inquiries can be directed to the corresponding authors.
